# Heavy Metal-Resistant Filamentous Fungi as Potential Mercury Bioremediators

**DOI:** 10.3390/jof7050386

**Published:** 2021-05-14

**Authors:** Cristina L. Văcar, Enikö Covaci, Somsubhra Chakraborty, Bin Li, David C. Weindorf, Tiberiu Frențiu, Marcel Pârvu, Dorina Podar

**Affiliations:** 1 Department of Molecular Biology and Biotechnology, Babeș-Bolyai University, 1 Kogălniceanu St., 400084 Cluj-Napoca, Romania; cristina.vacar@ubbcluj.ro; 2 Centre for Systems Biology, Biodiversity and Bioresources (3B), Babeș-Bolyai University, 3-5 Clinicilor St., 400015 Cluj-Napoca, Romania; marcel.parvu@ubbcluj.ro; 3 Department of Analytical Chemistry, Babeș-Bolyai University, 11 Arany Janos St., 400028 Cluj-Napoca, Romania; eniko.covaci@ubbcluj.ro (E.C.); tiberiu.frentiu@ubbcluj.ro (T.F.); 4 Research Center for Advanced Analysis, Instrumentation and Chemometrics, Babeș-Bolyai University, 11 Arany Janos St., 400028 Cluj-Napoca, Romania; 5 Agricultural and Food Engineering Department, Indian Institute of Technology, Kharagpur 721302, India; somzcall@gmail.com; 6 Department of Experimental Statistics, Louisiana State University, Baton Rouge, LA 70803, USA; bli@lsu.edu; 7 Department of Earth and Atmospheric Sciences, Central Michigan University, 1200 S. Franklin St., Mount Pleasant, MI 48859, USA; weind1dc@cmich.edu; 8 Department of Taxonomy and Ecology, Babeș-Bolyai University, 44 Republicii St., 400015 Cluj-Napoca, Romania

**Keywords:** mercury, mycoremediation, heavy metal resistance, *Fusarium* sp., *Sarocladium* sp., biosorption, contaminated soil, bioremediation, biotechnology

## Abstract

Filamentous fungi native to heavy metals (HMs) contaminated sites have great potential for bioremediation, yet are still often underexploited. This research aimed to assess the HMs resistance and Hg remediation capacity of fungi isolated from the rhizosphere of plants resident on highly Hg-contaminated substrate. Analysis of Hg, Pb, Cu, Zn, and Cd concentrations by X-ray spectrometry generated the ecological risk of the rhizosphere soil. A total of 32 HM-resistant fungal isolates were molecularly identified. Their resistance spectrum for the investigated elements was characterized by tolerance indices (TIs) and minimum inhibitory concentrations (MICs). Clustering analysis of TIs was coupled with isolates’ phylogeny to evaluate HMs resistance patterns. The bioremediation potential of five isolates’ live biomasses, in 100 mg/L Hg^2+^ aqueous solution over 48 h at 120 r/min, was quantified by atomic absorption spectrometry. New species or genera that were previously unrelated to Hg-contaminated substrates were identified. Ascomycota representatives were common, diverse, and exhibited varied HMs resistance spectra, especially towards the elements with ecological risk, in contrast to Mucoromycota-recovered isolates. HMs resistance patterns were similar within phylogenetically related clades, although isolate specific resistance occurred. *Cladosporium* sp., *Didymella glomerata*, *Fusarium oxysporum*, *Phoma costaricensis*, and *Sarocladium kiliense* isolates displayed very high MIC (mg/L) for Hg (140–200), in addition to Pb (1568), Cu (381), Zn (2092–2353), or Cd (337). The Hg biosorption capacity of these highly Hg-resistant species ranged from 33.8 to 54.9 mg/g dry weight, with a removal capacity from 47% to 97%. Thus, the fungi identified herein showed great potential as bioremediators for highly Hg-contaminated aqueous substrates.

## 1. Introduction

Heavy metals (HMs) are harmful for biological systems when present in excess and can often accumulate, as they are non-biodegradable [1]. HM contamination is an inconspicuous and persistent issue threatening environmental safety and human health [2]. Anthropogenic activities substantially contribute to worldwide HM dispersal in soil, water, and the atmosphere [3]. Estimates indicate up to 2.5 million potentially contaminated sites in Europe alone [4,5,6]. Municipal and industrial waste treatment and disposal, and mining activities, are among the major sources of HMs contamination in European soils [5]. Over the past five decades, single metal pollution of surface water has shifted to mixed metal pollution, and an increasing number of metals are surpassing World Health Organization (WHO) and US Environmental Protection Agency (USEPA) standard threshold concentrations [7]. Mercury (Hg) is of special concern for human health, as it is highly mobile in the environment and tends to bioaccumulate and further biomagnify through food webs [8,9,10]. Industrial and wastewater discharge, mining, coal combustion, and chloralkali industries were estimated to have increased the elemental Hg (Hg^0^) release into the atmosphere, above natural levels, by 1- to 4.5-fold, eventually increasing redeposition rates [11,12,13]. Elevated concentrations of Hg^0^, constantly generated by chloralkali plants, endanger the stability of ecosystems and human health in areas extending several km away from the point source [14,15,16,17,18,19]. Moreover, Hg atmospheric residence time and currents promote its global distribution, spreading the contamination to unspoiled areas [10].

Transboundary Hg contamination threatens human and ecosystem health, which is why coordinated preventive actions are undertaken globally to monitor and reduce its spread, and to regulate Hg usage and waste disposal [20]. Areas around point sources of HM pollution require urgent interventions to reclaim environmental safety and reduce adverse effects on human health. However, conventional techniques such as vitrification, incineration, excavation of landfill, soil washing, solidification, or electro-kinetic system stabilization imply high costs, intensive labor, subsequent dispersal, and irreversible changes in soil quality, structure, and essential activities of native microbiota [21,22,23]. Therefore, identifying low cost and less invasive technologies is necessary for addressing the remediation of HM-contaminated sites. In situ bioremediation approaches, i.e., biostimulation, biosparging, bioventing, and bioaugmentation [24,25,26], or microorganisms-assisted phytoremediation [27,28,29], have been recently considered for the decontamination of HMs contaminated soils. The use of bioremediation technologies can prevent secondary HMs dispersal and unnecessary human exposure can be avoided.

Mycoremediation is a type of bioremediation that employs fungi for removal, degradation, or toxicity reduction of numerous contaminants from various substrates [30]. Filamentous fungi exhibit essential characteristics that recommend them as effective HM bioremediation agents. Fungal cells have high surface area with excellent HM-binding properties due to negative charges of functional groups present in cell wall components [31,32]. Additionally, fungi possess multiple antioxidant systems, metal transporters, metal-buffering molecules, metal-transformation enzymes, vacuolar sequestration abilities, and secrete metal-precipitating compounds; however, few mechanisms have been characterized in filamentous fungi [25,33,34,35]. Not only are they cosmopolitan microorganisms, but they are also frequently more resilient than bacteria in metalliferous soils [36,37,38] and have the ability to colonize porous matrices with their hyphae networks. Research on fungi in relation to Hg has indicated several potential mycoremediation candidates, all of which have been isolated from HM-contaminated backgrounds [39,40,41]. The need for HM resistant fungi autochthonous to a contaminated site has been recently emphasized for in situ mycoremediation approaches [31,42,43,44]. The advantages are obvious: these isolates are already adapted to site-specific conditions, especially to competition within local communities, and are equipped with resistance/tolerance mechanisms that may account for toxicity reduction or removal of HMs at the site [31,42,43,44]. Lab-scale and greenhouse experiments validate soil HM mycoremediation performance [24,31,39]. Similarly, effective recovery of HMs from contaminated water by fungi as biosorbents has also been demonstrated at small-scales [45,46,47,48]. Fungal biosorbents were extensively studied by previous researchers [46,47,48,49,50,51,52]. However, the complexity of the biosorption process, development, and cost effectiveness of adequate techniques for large-scale application await further research and deliberation prior to industrial transfer [53,54]. Moreover, potential mycoremediators are suitable for studies that would broaden the understanding of molecular HM resistance mechanisms, and would eventually unlock new decontamination biotechnologies.

This study aimed to explore the diversity and the bioremediation potential of heavy metal resistant fungi. We hypothesized that fungi native to a historically contaminated site must have exceptional HM resistance that can be exploited for the development of bioremediation. Thus, the objectives were to: (i) isolate filamentous fungi from the rhizosphere of plants resident on a former chloralkali facility, and to identify them via internal transcribed spacer region; (ii) establish the isolates’ HMs resistance spectrum (Hg, Pb, Cu, Zn, and Cd) and degree in vitro, in solid media, via tolerance index (TI) and minimum inhibitory concentration (MIC), respectively; and (iii) assess the Hg^2+^ remediation potential of HM highly resistant fungal isolates by quantifying their live biomasses removal (%) and biosorption capacity (mg/g dry weight) in aqueous solution. Additionally, to investigate the functional profile of the community, the HMs resistance patterns were correlated with fungi phylogeny by hierarchical clustering, principal component, and k-means cluster analyses.

## 2. Materials and Methods

### 2.1. Site Description and Soil Sampling

**Figure 1:**
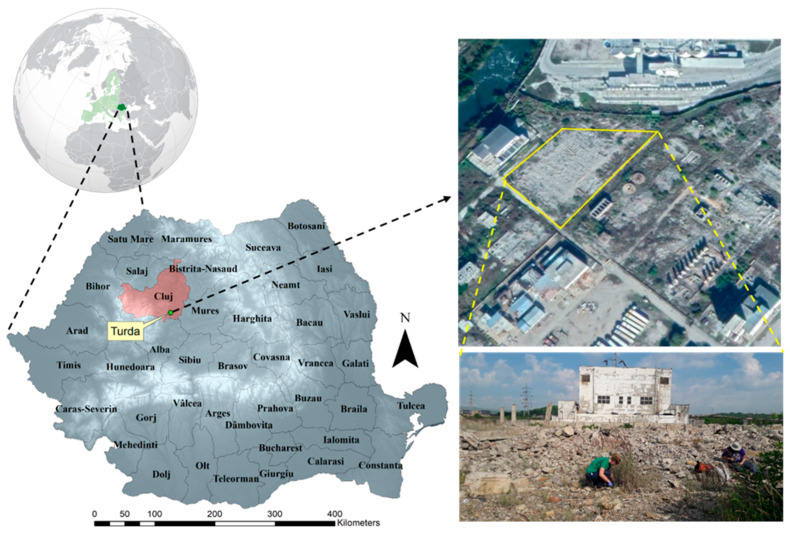
Location of the sampling site in Turda, Romania, in Europe.

The study site is located at a former chloralkali plant (N = 46.557192°, E = 23.781689°) in Turda, Cluj County, Romania, in the vicinity of the Arieș river basin [55,56] (Figure 1). The site was chosen for its historical Hg contamination—about 50 years of activity—since Hg was used at the cloralkali plant from 1958 as a cathode in the process of NaCl/KCl electrolysis to obtain NaOH/KOH and chlorine. Later, in the 1980s, plant utilities were extended to produce Cu and Zn pesticides, Ca(ClO)_2_, and other inorganic salts. Once plant activity ceased (late 1990s), the industrial facilities were demolished and abandoned. To date, the area was declared contaminated; yet, 25 years post decommission, no remediation solutions have been adopted [14,17,57]. The area is characterized by the Köppen climate classification as Dfb (warm humid continental), with a mean annual temperature of 9 °C and mean annual precipitation of 571 mm [58]. Geologically, soils of the area are mostly luvisols derived from clay migration, with the formation of eluvial and illuvial horizons [59]. However, soils sampled as part of this study were classified as Orthents [60], as they were rife with anthropogenic disturbance.

Sampling was performed in June 2018, on the location of the former electrolysis building where Hg was used as a cathode. The area is about 800 m^2^ and is mainly covered by demolition debris. Flora and its diversity on the site are scarce. A few species belonging to Brasicaceae, Asteraceae, Fabaceae and Poaceae families were present at the periphery of the demolition residues. Sampling included all the plant species present at the site, without aiming for Hg hot spots. Twenty rhizosphere soil samples were collected in plastic bags using a stainless-steel hand trowel, from the roots of individual plants scattered around the site’s perimeter, at a depth of 5–25 cm [61]. Soil samples were stored at 4 °C and individually processed for microbial culturing and for elemental analysis within 24 h upon collection.

### 2.2. Reagents, Stock Solutions and Certified Reference Materials

Single element stock solutions of 0.125 mol/L Hg^2+^ and 1 mol/L Cd^2+^, Cu^2+^, Pb^2+^, Zn^2+^ were prepared from HgCl_2_ (≥99.5%), 3CdSO_4_·8H_2_O (≥99.0%), CuSO_4_ (≥99.0%), Pb(NO_3_)_2_ (≥99.0%), and ZnSO_4_ (≥99.0%) (Sigma-Aldrich, Munich, Germany). The solutions were sterilized through Millex^®^ Syringe Filter (Merck, Darmstadt, Germany), 0.22 μm pore size, and utilized for assessment of fungal HM resistance spectrum in Czapek-Dox agar medium (Formedium, Hunstanton, UK). An aqueous solution of 100 mg/L Hg^2+^ was prepared and utilized to assess fungi removal and biosorption potential from supernatants.

Reagents used for supernatant sample preparation and calibration solution dilutions were HCl 37% for Hg determination (≤10^−4^% Hg), NaOH (>98%), and NaBH_4_ for analysis, utilized for the preparation of the reaction medium (3% *v*/*v* HCl), and 0.3% (*m*/*v*) of NaBH_4_ stabilized in 0.2% NaOH (*m*/*v*), needed for Hg determination by cold vapor high-resolution continuous source quartz furnace atomic absorption spectrometry (CV-HR-CS-QFAAS). Standard solutions in the range of 0–10 μg/mL Hg^2+^ (*n* = 9) in 3% (*v*/*v*) HCl, prepared from inductively coupled plasma (ICP) standard solution of 1000 μg/mL Hg^2+^ in 10% HNO_3_ (Merck, Darmstadt, Germany) were used for CV-HR-CS-QFAAS calibration [17]. A blank solution (3% (*v*/*v*) HCl) was used as dilution agent and for the evaluation of the limit of detection (3σ criterion, *n* = 11). The content of Hg^2+^ in supernatant at each sampling time point was determined using a dilution to fit the calibration curve in 3% HCl (*v*/*v*). Double distilled water was utilized throughout the study for solution preparation and vessel cleaning.

To ensure the accuracy of elemental analysis in fungal supernatants and soil samples, the following certified reference materials were used: ERM-CA713 wastewater certified reference material (Institute for Reference Materials and Measurement, Geel, Belgium) and NIST 2711a-Montana II soil (National Institute of Standards and Technology, Gaithersburg, MD, USA) [62].

### 2.3. Elemental Analyses and Quality Control

The rhizosphere soil samples were analyzed for Hg, Pb, Cu, Zn, and Cd content by portable X-ray fluorescence (PXRF) with a Vanta series handheld spectrometer (Olympus, Waltham, MA, USA) [63]. The samples were dried at 20 °C until at constant weight, then ground to fine powder with an agate mortar and a pestle. Samples were massed to infinite thickness on a Prolene thin film placed directly over the instrument aperture mounted in a portable test stand configuration [64]. The instrument was initialized with a 316 stainless steel alloy and operated on line power (115 VAC) at 10–40 keV for 45 s/beam in *Geochem Mode*. Quality assurance was afforded via scanning NIST 2711a-Montana II soil for the relevant metals of this study: Hg, Pb, Cu, Zn, and Cd. Additionally, a correction factor (CF) for each metal was calculated and applied to the raw detected values a priori [62,65]; data on recovery values and applied CFs are given in Table S1. Further, ecological risk index (RI) was calculated for the metals considered under this study [66]. Pre-industrial reference values used were from the natural background content of upper continental crust [67,68].

Determination of Hg concentration remaining in supernatants after fungi biosorption was carried out by CV-HR-CS-QFAAS using the ContrAA 300 spectrometer (Analytik Jena, Jena, Germany), equipped with the HS55 batch chemical system generator, in accordance with the manufacturer recommendations. Volumes of 5 mL calibration solution/sample were pipetted into the reaction cell, where Hg cold vapors were generated with 3.5 mL 0.3% (*v*/*v*) NaBH_4_ solution stabilized in 0.2% (*v*/*v*) NaOH, pumped via the peristaltic pump for 13 s. Mercury vapors were transported from the reaction cell into the 150 ± 10 °C heated quartz tube with a flow rate of 6 L/h Ar, where they absorbed the Xe lamp emitted light at 253.652 nm. Signals were processed as peak height from transient signals, registered for a time of 33 s in a narrow wavelength range (±0.2 nm), and peak heights were calculated based on 5 pixels. The CV-HR-CS-QFAAS method was validated for mercury determination, with a detection limit of 0.067 μg/L Hg^2+^ and a recovery in the range of 97 ± 9% in ERM-CA713 (certified value 1.84 ± 0.11 μg/L, found value 1.79 ± 0.16 μg/L for *n* = 5 and 95% confidence level).

### 2.4. Fungal Isolation and Heavy Metals Resistance Assays

Refrigerated soil samples were sieved through a 2 mm stainless steel sieve and further processed by a routine serial dilution method on Czapek-Dox agar medium, often used for recovery of fungi from HM-contaminated soils [69,70,71,72]. The HM resistant fungal isolates were selected upon cultivation on Czapek-Dox agar medium supplemented with single metals (Hg, Pb, Zn, Cu, and Cd), as described in Section 2.2. Metals were added to the autoclaved media and cooled at ~40 °C from single element stock solutions. The concentration (mg/L) of each metal, 10 Hg^2+^, 518 Pb^2+^, 63.5 Cu^2+^, 490 Zn^2+^, and 56.2 Cd^2+^, allowed for discrimination between sensitive and resistant isolates, and were chosen considering commonly used concentrations in previously published studies [43,73,74]. Medium without metal solution served as control. The plates were centrally inoculated and incubated at 28 °C for 7 days. Two diameters were recorded and used for the generation of the tolerance index (TI). The TI represents the ratio between the mean of two fungal growth diameters in metal supplemented and control plates [75]. TIs of each fungal isolate were determined for each of the studied metals. The HM resistance was classified based on TIs as follows: 0—sensitive, 0 > TI > 0.29—very low resistance, 0.30 > TI > 0.49—low resistance, 0.50 > TI > 0.69—moderate resistance, 0.70 > TI > 0.89—high resistance, and >0.90—very high resistance, adapted from Oladipo et al. [44].

A set of seven fungi was selected for the minimum inhibitory concentration (MIC) assay, based on their superior Hg and multiple HMs resistance as assessed by TIs. Each isolate was tested for the metals it previously exhibited resistance towards. Potato dextrose agar (PDA) medium was used for comparison with MIC values previously reported [43,73,74]. The MIC of each isolate was assessed in triplicate on PDA medium supplemented with increasing concentrations of Hg^2+^, Pb^2+^, Cu^2+^, Zn^2+^, or Cd^2+^, adapted for an individual isolate’s growth. Plates were centrally inoculated with a 6 mm diameter fungal disk excised from the edge of 7-day-old pre-grown cultures on PDA. The MIC was recorded upon incubation at 28 °C for 10 days.

### 2.5. Species Diversity and Phylogeny

Isolates that exhibited resistance above moderate (TI ≥ 0.5) for at least one of the investigated metals were identified based on the internal transcribed spacer (ITS) molecular marker. The gDNA was extracted using the Animal and Fungi DNA preparation kit (Jena Bioscience, Jena, Germany, #PP-208L), as per manufacturer instructions, and used as a template for ITS amplification. The ITS region was amplified using the ITS1F (5′-CTTGGTCATTTAGAGGAAGTAA-3′) and ITS4 (5′-TCCTCCGCTTATTGATATGC-3′) primers [76,77], and the DreamTaq Green PCR Master Mix (2×) (ThermoFisher Scientific, #K1081). The PCR products were confirmed by electrophoresis in 1% agarose gel, before purification with GeneJET PCR purification kit (ThermoFisher Scientific, #K0702). Sequencing was performed with ITS1F primer with an ABI 3730xl system based on capillary electrophoresis sequencing (Macrogen, Amsterdam, The Netherlands). Sequences were examined and manually edited using Chromas (Version 2.6.6, 2018, Technelysium Pty Ltd., South Brisbane, Queensland, Australia). The curated sequences were used as queries in the BLASTn, at NCBI public databases. The species corresponding to the entry with the highest total score was attributed to the inquired sequence. The GenBank accession numbers of the fungal isolates are given in Table S2. Additionally, the ITS sequences were subjected to multiple ClustalW alignment, followed by a 1000 replicate bootstrap analysis to build a neighbor-joining tree in MEGA X software [78], then processed for visualization by iTOL [79]. The ITS sequence from *Allomyces arbuscula*, AY997028.1, was used as an outgroup because it belongs to the Chytridiomycota phylum, an early diverging clade within Fungi. The phylogeny was used to explore similarities among specific taxa and metal resistance patterns.

### 2.6. Hg^2+^ Biosorption from Aqueous Solution

Hg^2+^ removal and biosorption capacities of five distinct fungal species with superior Hg resistance, were established in aqueous solution supplemented with 100 mg/L Hg^2+^, similar to Zafar et al. [32]. Water was selected, as our experiments in Luria Bertani and PDB media, that were used by other authors in similar conditions [39,80], resulted in important media-driven Hg loss. Seven-day-old PDA fungal cultures were used to prepare the spore suspensions (10^6^ spores/mL). Thirty mL liquid PDB medium was inoculated in triplicate at a final concentration of 10^4^ spores/mL, and incubated for 7 days at 28 °C, 120 r/min. The developed fungal biomass was recovered by vacuum filtration through MF-Millipore™ MCE membrane filters (Merck, Darmstadt, Germany), 0.45 μm pore size. Washed fungal biomass was added to a 100 mg/L Hg^2+^ aqueous solution and incubated for 2 days at 28 °C, 120 r/min. The solution without fungal biomass served as control. Fungal biomass removal capacity was monitored by determination of Hg^2+^ concentration in supernatant, free of cells, collected at 0.5 h, 2 h, 6 h, 12 h, 24 h, and 48 h. At each time interval, the fungal cultures were centrifuged at 4300 r/min for 15 min. Aliquots of 2 mL supernatant were further centrifuged at 13,000 r/min for 10 min to obtain supernatant free of cells. The concentration of Hg^2+^ in the supernatant free of cells was determined by the CV-HR-CS-QFAAS method. At 48 h, the biomass was recovered by vacuum filtration through MF-Millipore™ MCE membrane filters, with 0.45 μm pore size, then dried at 33 °C until at a constant weight. The biosorption capacity (*Q*) was calculated with Equation (1) [81]:

*Q* = (*C*_0_ − *C*)*V*/*m*, (1)

where *C*_0_ (mg/L) and *C* (mg/L) are the Hg^2+^ concentrations at 48 h in control and in fungal supernatant solutions, respectively; *V* (L) is the volume of the solution, and *m* (g) is the dry weight (d.w.) of biomass.

### 2.7. Statistical Analyses

One-way analysis of variance (ANOVA) followed by Tukey Pairwise Comparison with 95% confidence were used to assess differences between means (Minitab^®^ 17.1.0, State College, PA, USA). Matrix-plot was applied to individual TIs in Past 4.03 software [82]. Hierarchical clustering analysis (HCA) of individual TIs, principal component analysis (PCA), and k-means cluster analysis of genera mean TIs, using the ‘prcomp’ package after scaling, were executed in R version 3.6.2 [83] to compare HMs resistance patterns with fungi phylogeny. To determine the optimal number of clusters (k), the average silhouette approach was used. Library ‘cluster’ was used in R to calculate silhouette information, while the ‘kmeans’ function was used for k-means clustering.

## 3. Results

### 3.1. Heavy Metal Concentrations in Rhizosphere Soil

The basic statistics of variation range and distribution of Hg, Pb, Cu, Zn, and Cd in the 20 rhizosphere soil samples are presented in Table 1. The severity of Hg contamination was confirmed, as values exceeded 3.5 to 200 times the industrial soil threshold intervention value. With respect to Pb, 18 out of 20 rhizosphere soil samples exceeded up to 3.2 times the industrial soil threshold alert value. Ten out of 20 samples exceeded up to 6 times the industrial soil threshold intervention value for Cu, while five exceeded the industrial soil threshold alert value for Zn. Data demonstrate an asymmetric distribution of Hg, Cu, and Zn around the mean, i.e., with positive skewness and leptokurtic distribution (kurtosis > 3). With respect to Pb, the distribution is normal and platykurtic. Therefore, the median values were considered to evaluate the ecological risk index (RI) per Hakanson [66]. Although the concentrations of each element varied within the sampling site, very high ecological risk was found for Hg (RI = 390,000 > 600), moderate risk for Pb (RI = 189 > 150), and low risk for Zn, Cu, and Cd for the rhizosphere soil samples.

### 3.2. Identity and Heavy Metal Resistance Phylogenetic Patterns of Isolated Fungi

**Figure 2:**
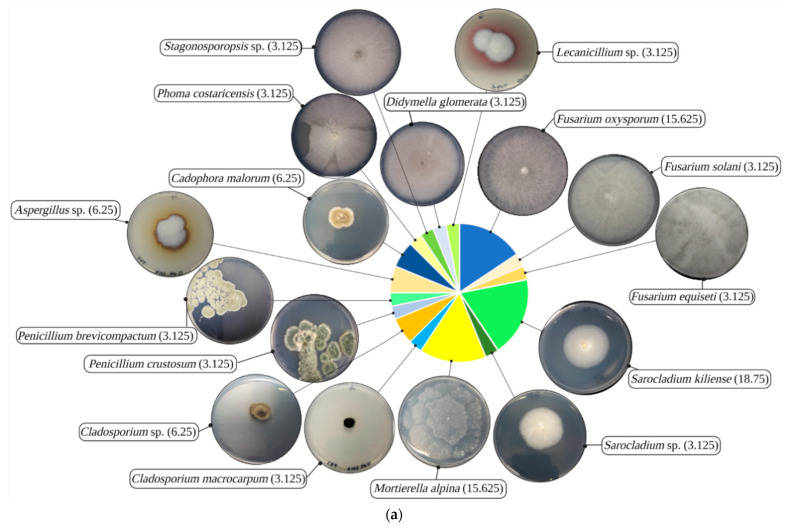

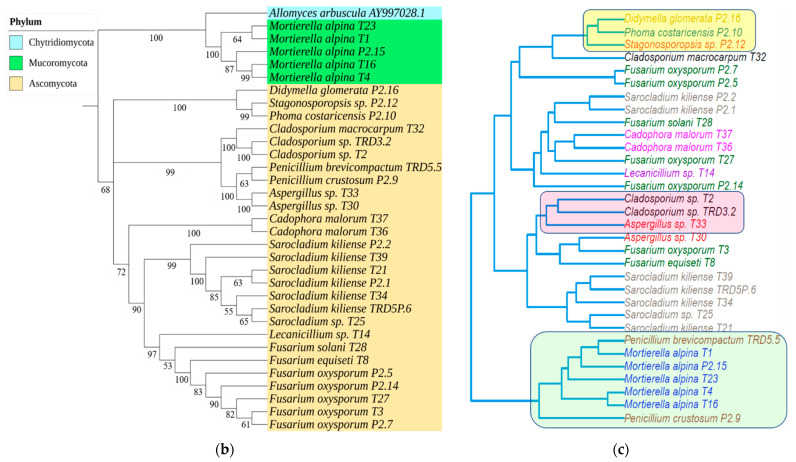
(**a**) Composition of the isolated fungal community and the phenotypes of 7-day-old cultures on Czapek-Dox agar, with percentages of species (BioRender, exported on 8 April 2021); (**b**) species phylogeny (neighbor-joining tree) based on internal transcribed spacer sequences (*Allomyces arbuscula* AY997028.1 was used as an outgroup); and (**c**) dendrogram of hierarchical clustering analysis using the individual tolerance indices.

The diversity and species abundance of 32 culturable fungal isolates with moderate or higher resistance (TI ≥ 0.5) for at least one HM are illustrated in Figure 2a. Based on their ITS sequences, 25 isolates were identified at the species level, while seven isolates were resolved at the genus level. Ascomycota was the most abundant and diverse phylum recovered as culturable and metal resistant fungi from the investigated site. The isolated Mucoromycota representatives recovered were less diverse, belonging solely to *Mortierella alpina* (Figure 2b). The fungal community was delineated by Mortierellales, Pleosporales, Capnodiales, Eurotiales, Heliotiales, and Hypocreales orders. Hierarchical clustering analysis (HCA) of individual TIs for Hg, Pb, Cu, Zn, and Cd was executed to assess whether HMs resistance patterns overlap fungi phylogeny (Figure 2c). Representatives of Pleosporales, here *Didymella glomerata*, *Phoma costaricensis*, and *Stagonosporopsis* sp. HCA sub-cluster (yellow box), displaying a high and very high Hg and Pb-resistance pattern, reflected their phylogeny. Additionally, *Cladosporium* sp. (Capnodiales) and *Aspergillus* sp. (Eurotiales) sub-cluster (pink box), displaying a moderate to very high Hg resistance pattern, coincided with their phylogenetic relationship. However, *Penicillium* sp. (Eurotiales) were clustered with *Mortierella* sp. (Mortierellales) (green box), having a moderate Zn resistance pattern and sensitivity for the other contaminants, regardless of their phylogenetic positions. *Cadophora malorum* (Heliotiales), *Sarocladium*, *Fusarium*, and *Lecanicillium* (Hypocreales) clustering, presenting diverse HMs resistance patterns, resembled their phylogenetic group, although they were fragmented into separate clusters. These observations show that, in heavily Hg-contaminated soil, Ascomycota is the dominant phylum, and that HM resistance patterns within the fungal community overlap phylogenetically in general.

**Figure 3:**
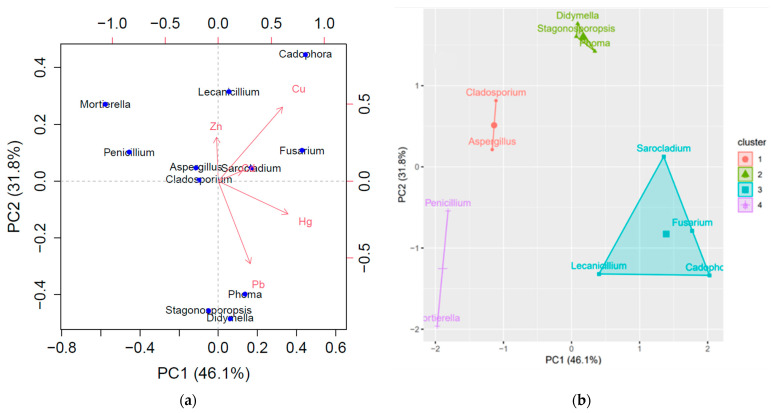
(**a**) Principal component (PC) analysis biplot and (**b**) k-means cluster plot, using the first two principal component scores, applied to the tolerance indices mean of each genus.

PCA analysis was applied to the mean TIs of each fungal genus for each of the HM assessed to evaluate the community response against metal stress (Figure 3a). The leading two PCs explained ~78% of the total variance. Examining the loading weights, it was clear that PC1 was dominated by the sum of Hg and Cu, PC2 was dominated by the difference between (Cu + Zn) and Pb, while Cd was not strongly associated with the remaining variables. Cu and Pb were almost uncorrelated, while Zn and Pb were negatively correlated. From the PCA scores, it was clear that *Cadophora* had high levels of Cu resistance and moderate levels of Hg and Zn resistance. The large PC1 value of *Fusarium* indicated its high level of Hg and Cu resistance. The *Sarocladium* position corresponded to a moderate Hg and Cd resistance. Both *Stagonosporopsis* and *Didymella* were at the bottom of the plot, indicating that they likely had high Pb resistance and a low resistance for Cu and Zn. Moreover, *Mortierella* appeared in the leftmost corner of the plot, which implied that it had a low resistance to Hg and Cu and moderate Zn resistance. Both *Aspergillus* and *Cladosporium* were in the center, indicating average levels of HM resistance.

The average silhouettes vs. k plot, when using the genera TIs mean, rendered four optimum clusters that were plotted in the k-means cluster plot (Figure 3b). Cluster 1 defines a moderate or higher Hg-resistance pattern. Cluster 2 specifies a moderate or higher Hg and very high Pb resistance pattern. Cluster 3 mostly designates a high and very high Hg resistance pattern, accompanied occasionally by moderate or higher Cd, Cu, or Pb resistance. Cluster 4 represents a sensitive pattern for the contaminants present at the investigated site. The clustering patterns contoured are consistent with those in the PCA plot and HCA diagram. As statistical analyses indicated a degree of relatedness between HMs resistance patterns and phylogenetic relationships, taxonomic group characteristics (i.e., structure and composition of cell walls, type, and quantity of secreted molecules) likely reflect common advantages for representatives of a certain cluster. However, particular characteristics of certain species or isolates contextualize the scattering effect.

### 3.3. Fungal Heavy Metal Resistance Spectrum

**Figure 4:**
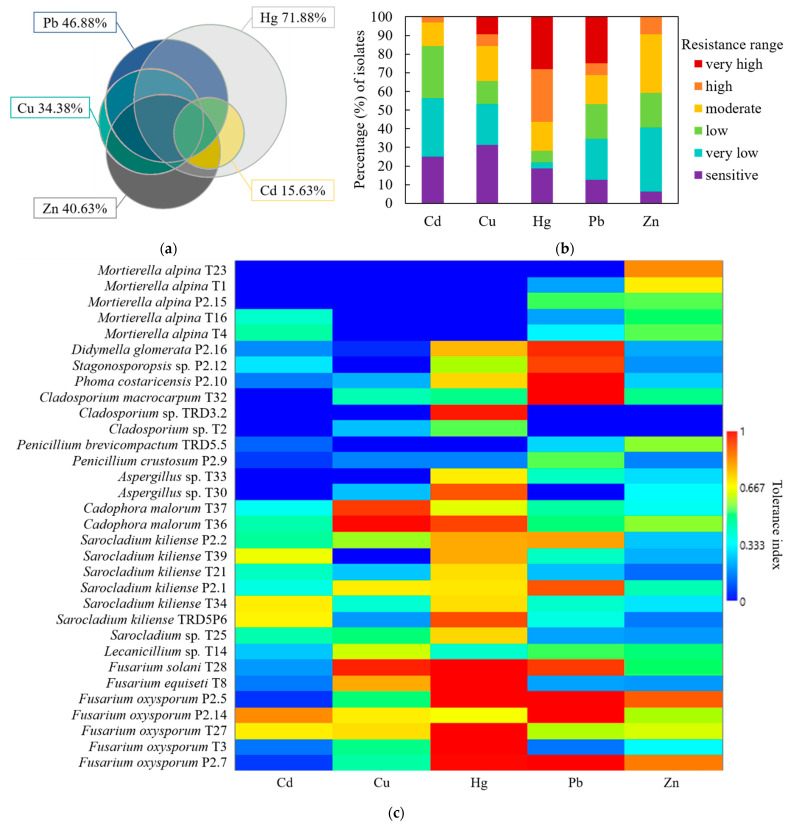
Fungi community, illustrated as percentages of isolates (**a**) with single and multiple metals resistance (TI ≥ 5), (**b**) according to tolerance index ranges, and (**c**) with matrix-plot of individual TI.

The 32 isolated fungi were investigated on Czapek-Dox agar medium for their ability to withstand stress induced by a single element solution of 10 Hg^2+^, 518 Pb^2+^, 63.5 Cu^2+^, 490 Zn^2+^, and 56.2 Cd^2+^ (mg/L). The percentage of isolates that displayed moderate or higher resistance (TI ≥ 0.5) for one or more HMs is illustrated in Figure 4a. Single element resistance was detected for 40.63% of isolates, while 59.37% withstood stress induced independently by multiple elements. Cadmium and Cu resistance was always associated with that for Hg, Pb, or Zn. Single resistance was observed primarily for Hg (18.75%), followed by Zn (15.63%) and Pb (6.25%). Since the sampling site is heavily Hg-contaminated, it was not surprising that 71.88% of the fungal isolates exhibited moderate or higher resistance for this element. Furthermore, 53.13% of the isolates were Pb, Cu, Zn, or Cd-resistant, complementary to Hg.

The proportion of fungal isolates classified according to their resistance degree for each element assessed is shown in Figure 4b. Most of the isolates, i.e., 81.25%, displayed an Hg-resistance phenotype, predominantly (56.25%) in the very high and high ranges. In addition to very high and high Hg resistance, 9.38% of the isolates were also very highly Pb-resistant. Isolates’ resistance for Zn, Cu, and Cd was less frequent and/or ranged dominantly from moderate to very low. Only 3.13% and 15.63% of isolates displayed high and very high resistance towards Cd and Cu, respectively. The mean Hg TIs of fungal isolates was 0.62, higher than for Pb (0.5) and significantly higher (*n* = 32, *p* < 0.001) than for Zn (0.4), Cu (0.35), and Cd (0.27). The HMs’ occurrence and availability in rhizosphere soil at the investigated site likely represents a selection factor towards a fungal community equipped with effective resistance mechanisms. Since Cd was not detected by PXRF in the rhizosphere soil samples, whereas Zn and Cu are micronutrients constantly uptaken by plant roots, it is probable that these elements had a secondary contribution in shaping the fungal community composition. The Hg selective pressure on the investigated fungi was demonstrated based on their responses to stress induced by HMs: prevalence of very high and high Hg-resistant phenotypes and specific resistance, primarily to Hg.

The matrix-plot of individual TIs suggestively illustrates isolates’ identity in relation to their resistance spectrum and their resistance degree against the assessed contaminants (Figure 4c). *Fusarium* and *Sarocladium* genera shared Hg resistance, although to different degrees, mostly very high and high, respectively. In addition to Hg, *Fusarium* isolates often had very high Pb and high to moderate Zn resistance, while *Sarocladium* isolates exhibited moderate to very low Cd resistance. For these isolates, the detoxification mechanisms of Cd are likely the same as for Hg, since moderate or higher Cd resistance was always associated with that of Hg. Only one isolate of *Cladosporium* and of *Aspergillus* genera displayed very high Hg-resistance. The *Cadophora* genus commonly had very high Cu resistance, although the isolates had different degrees of Hg resistance. Overall, the HMs resistance spectrum is similar among isolates of the same genus, although to different degrees of resistance. Notably, *Mortierella alpina* isolates were Cu- and Hg-sensitive, but mostly moderately Zn-resistant, and rarely low Cd-resistant. Isolates with superior HMs resistance effectively act as in situ ecological engineers, reducing elements bioavailability they create habitable microconditions for other sensitive microorganisms.

The spectrum of HMs resistance within the fungal community was primarily directed to Hg, followed by Pb, Zn, Cu, and Cd. This observation indicated that elements present at the site exerted selective pressure on the fungal population, in accordance with the ecological risk index. Moreover, Hg resistance of fungi was often coupled with other HMs, suggesting great potential for the development of bioremediation of multiple-contaminated substrates. Additionally, it was observed that, although HMs resistance spectra are similar between closely related species, the degree of resistance can be isolate-unique.

**Figure 5:**
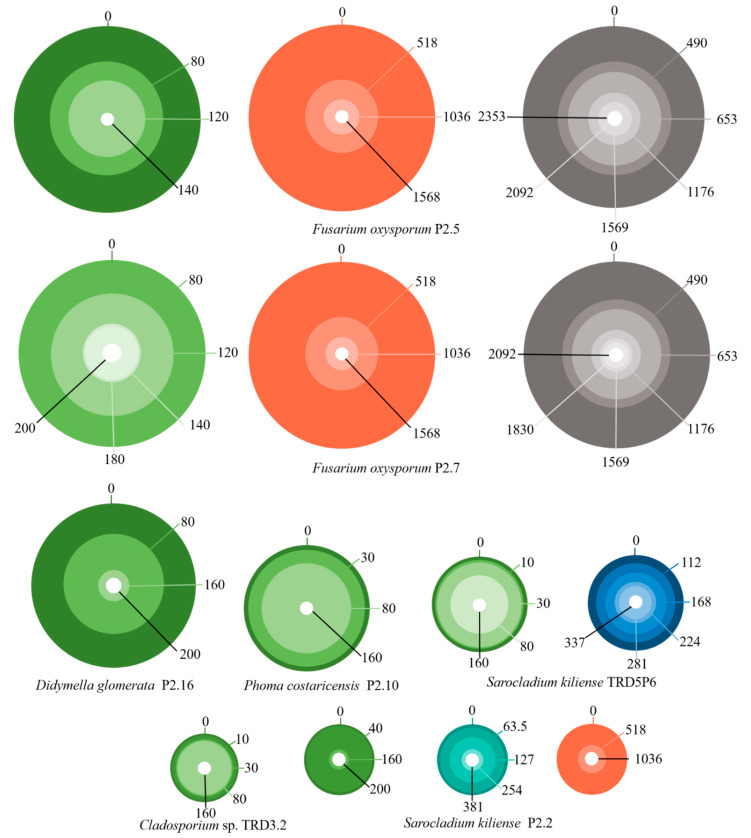
Representation of the minimum inhibitory concentration assay (mg/L) for investigated fungal species: Hg^2+^ (green), Pb^2+^ (red), Zn^2+^ (gray), Cd^2+^ (dark blue), Cu^2+^ (turquoise).

The MIC for seven fungal isolates that displayed very high to moderate resistance for one or more metals was established in PDA (Figure 5). The dimension of the outer circle is proportional to fungal growth at 10 days on the control plates, while the inner circles represent growth inhibition induced by the gradually increasing concentrations of tested metal ions (mg/L), displayed clockwise. The central white dot represents the completely inhibited fungal growth. The MIC value is the mean of at least three independent measurements (Table S3). *Fusarium oxysporum* P2.5 and P2.7 isolates had MIC values (mg/L) of 140 and 200 for Hg, 2353 and 2092 for Zn, and 1568 and 1568 for Pb, respectively. *Phoma costariensis* P2.10 and *Cladosporium* sp. TRD3.2 had Hg MIC values of 160, while *Didymella glomerata* P2.16 of 200. *Sarocladium kiliense* P2.2 presented MIC values of 200 for Hg, 1036 for Pb, and 381 for Cu, while *S. kiliense* TRD5P.6 of 337 and 160 for Cd and Hg, respectively. Upon this study, *Fusarium oxysporum* P2.5, *Sarocladium kiliense* TRD5P.6, *Cladosporium* sp. TRD3.2, *Phoma costaricensis* P2.10, and *Didymella glomerata* P2.16 were selected for the Hg^2+^ removal and biosorption assay from aqueous solution, as they represent distinct species. 

### 3.4. Removal and Biosorption Potential of Hg^2+^ from Aqueous Solution

**Figure 6:**
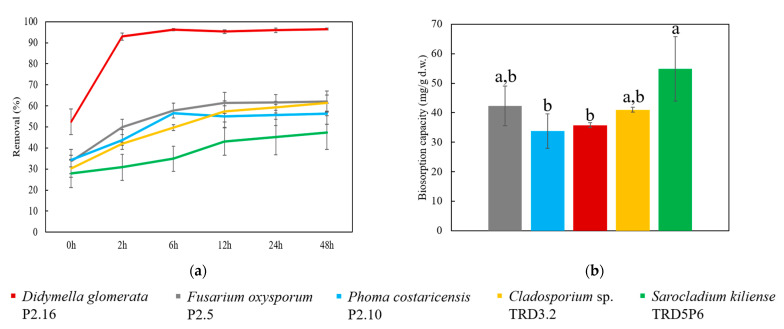
Mercury biosorption potential of five fungal isolates’ live biomasses from 100 mg/L Hg^2+^ aqueous solution over 48 h, 120 r/min: (**a**) removal and (**b**) biosorption capacity; the values represent means, the error bars represent confidence intervals (*n* = 3, 95% confidence level) and bars with different letters are significantly different (confirmed by Tukey test, *p* < 0.017).

The removal and biosorption potential of the live biomasses of five fungal isolates belonging to distinct species was established in 100 mg/L Hg^2+^ aqueous solution over 48 h contact time under shaking conditions (120 r/min) (Figure 6). The amount of Hg^2+^ ranging between 28% and 52% was removed by the fungal biomasses in 30 min. *Didymella glomerata* P2.16 live biomass performed 93% removal after 2 h of incubation, and the equilibrium was almost reached within 12 h by all isolates. At the end of the incubation period, the removal capacity of live biomasses was: 97 ± 0.4% (*D. glomerata*) > 62 ± 5.1% (*F. oxysporum*) ≈ 61 ± 3.9% (*Cladosporium* sp.) > 56 ± 5.0% (*Phoma costaricensis*) > 47 ± 8.0% (*S. kiliense*) (mean ± 95% confidence level, *n* = 3). The maximum removal capacity of *D. glomerata* biomass was significantly greater (*p* < 0.0001) than the other tested species. This was attributed to the highest biomass developed from spores in the PDB fungal cultures compared to other species. The potential of *D. glomerata* to form the highest biomass can be further exploited for bioremediation, despite its lower biosorption capacity of 35.8 ± 0.8 mg/g d.w. compared to other isolates. Conversely, despite having the lowest removal capacity, *S. kiliense* exhibited the highest Q. This was due to it having the lowest biomass development among the fungal species. The Hg^2+^ biosorption capacity of the tested fungal biomasses ranged from 33.8 ± 5.8 for *P. costaricensis* to 54.9 ± 11 mg/g d.w. for *S. kiliense* (mean ± 95% confidence level, *n* = 3). It is likely that the differences in d.w. were due to species characteristic growth rates, although the biomasses were initiated using equivalent spore numbers.

## 4. Discussion

Even though sparingly investigated, fungal communities are consistently found to be more resilient than bacterial ones in HMs contaminated substrates [37,38,85]. Metal-resistant fungi, autochthonous to contaminated soils, show remarkable degrees of resistance towards HMs. Their bioremediation potential has been recently explored, i.e., HMs removal capacity from single/multi-metal solutions or contaminated soil bioaugmented with fungal consortia, and their ability to assist in phytoremediation of HM-contaminated soil [24,31,32,43,44,86,87,88]. Unique fungal diversity was identified in various HM-contaminated sites [24,31,43,44,87,88]. Similar investigations on bacterial populations reflect the same results [56,89,90,91]. These studies emphasize the need to investigate and make use of microorganism consortia adapted to the particular conditions of each location.

Flora and associated microbiota are appearing at the study site, despite of the severe Hg-contamination. Plants and rhizosphere inhabiting microorganisms must therefore possess mechanisms that allow their survival under extreme conditions. As such, their study for the development of environmental clean-up biotechnologies is timely. The current study investigated the diversity, the metal-resistance profile, and the potential for Hg bioremediation of filamentous fungi isolated from the rhizosphere of plant species on a former chloralkali facility. Exceedingly high concentrations of Hg, along with elevated concentrations of Pb, Cu, and Zn, were confirmed via PXRF in the analyzed rhizosphere soil samples. The Hg selective pressure acted primarily on the studied fungal community, while Pb, Zn, Cu, and Cd had secondary contributions. In general, the HM resistance patterns were shared by closer phylogenetically related isolates. This suggests that, for their survival under HM stress, fungi employ mechanisms characteristic to the taxonomic group they belong to. Beneficial mutations resulted from recombination events, upon repair of double strand breaks induced by HMs, or horizontal gene transfer events [92,93,94] can grant isolate-unique HM resistance. The diversity and abundance of fungi isolated herein are in accordance with previous data describing Ascomycota as dominant in both natural and HM-contaminated soils [36,38,69,95,96,97], and Mucoromycota as less frequently recovered from soil with multi-toxic elemental contaminations [69].

The ubiquity of the Ascomycota phylum may be due to several factors: filamentous morphology that enables extended hyphal growth, wind-dispersal, versatility in lifestyles, and a high number of genes related to stress tolerance which provide significant advantages in colonizing various niches [98]. Isolates of *Fusarium* and *Sarocladium* genera, the most frequently recovered from the investigated site, were commonly detected in soil samples associated with mining activities [37,73,99]. The cosmopolitan nature and versatility of these two ascomycetous genera explain their high incidence at the investigated site [100,101]. The preponderance of *Fusarium* and of other genera identified here (i.e., *Penicillium*, *Lecanicillium,* and *Phoma*) has also been reported in contaminated areas or substrates containing Cu, Pb, or Zn [71,95,102]. Concerning fungal diversity from a former Hg mining plant in Rudňany, Slovakia, *Cladosporium cladosporioides*, *Penicillium* spp., *Aspergillus* spp., and *Fusarium oxysporum* species were recovered [88]. However, the composition of fungal communities is site-specific, influenced by soil composition, properties, and selective pressure of HMs [37,71,95,99]. Additionally, the plant community composition, followed by local and seasonal climate variations, are important contributors in shaping fungal communities [103,104,105]. Herein, new species or genera are reported that were previously unrelated to Hg-contaminated sources: *Didymella glomerata*, *Lecanicillium* sp., *Fusarium solani*, *F. equiseti*, *Sarocladium* sp., *Penicillium crustosum*, *P*. *brevicompactum*, *Cadophora malorum*, *Phoma costaricensis*, and *Stagonosporopsis* sp. Most of the isolates belonging to these species exhibited very high and high Hg resistance.

*Mortierella alpina* isolates, representatives of Mucoromycota, were recovered infrequently from the rhizosphere soil samples, and exhibited sensitivity for the principal contaminant at the investigated site, Hg, and for Cu. *Mortierella* genus is known for establishing beneficial associations with plants, providing enhanced growth and stress tolerance against pathogens [106,107,108]. It is likely that plants are unable to select isolates with increased HM resistance, but may select certain species providing beneficial effects instead. Leguminous plant species on an Hg contaminated site recruit particular rhizobacteria species, irrespective of the strains’ useful traits or HM resistance [109]. Accordingly, plants may not distinguish between HM-resistant or -sensitive strains. Thus, it is possible that *Mortierella alpina* isolates were engaged by indigenous plant species in symbiotic relationships, due to their responsiveness to root exudates’ chemotaxis, irrespective of their inferior metal resistance pattern. However, some of the HM-resistant ascomycetes species isolated herein are known to be involved in plant diseases [101]. We presume that these communities perform in a successive manner, i.e., beneficial fungi colonizing plants’ roots grant protection against pathogens throughout the vegetation period, whilst hypothetically phytopathogenic ones thrive as saprotrophs on decaying plant material at the end of plants’ life cycle [110] while decreasing HMs’ bioavailability until the next season. Conceivably, symbiotic fungi should be considered for phytoremediation approaches assisted by fungal consortia, beside the HM resistant ones. They likely play an essential role, especially in the early stages of plant development.

Concerning the Cd, Cu, Pb, and Zn MIC values reported herein, comparable values were previously documented for *Aspergillus*, *Penicillium*, *Fusarium*, or unidentified isolates in Sabouraud dextrose agar or PDA media [32,43,74]. However, Hg MIC values recorded in this study were higher than previously reported for other fungal species in different types of media. *Aspergillus flavus* withstood up to 100 mg/L Hg in PDA [111], while *Rhodotorula mucilaginosa*, isolated from a sediment sample, was able to grow in the presence of 80 mg/L Hg yeast peptone dextrose liquid medium [112]. An Hg-resistant *Lecythophora* sp. isolate displayed an MIC of 84.5 mg/L Hg in LB broth [39]. *Penicillium* sp. DC-F11, isolated from multiple metals contaminated soil in a mining area, had a 60 mg/L Hg MIC value in LB agar [42]. Therefore, the analysis of MIC for the seven isolates highlighted a significant degree of resistance to Hg especially, in addition to the other assessed HMs.

Biomasses developed by fungal species isolated from Turda had superior Hg^2+^ biosorption capacity in aqueous solution, relative to values reported for related studies [80,88,112,113,114,115,116,117]. However, various fungal and fungal-derived types of biosorbents have previously been investigated for their Hg removal and Q, under widely variable experimental conditions [40,45,111,112,113,114,115,116,117,118]. Thus, a uniform comparison is unattainable for such heterogeneous conditions. Under similar conditions to those herein, i.e., wet biomass in 100 mg/L Hg^2+^ aqueous solution, reported Qs were 0.02 mg/g d.w. for an Hg-resistant *Trichoderma virens* isolate [80] and 336 mg/g d.w. for *Lentinus edodes*, with a 0.89 m^2^/g surface area [45]. Often, powdered fungal biomass was used as an Hg^2+^ biosorbent because of its higher surface area, relative to live biomass, i.e., Q of 10 and 27 mg/g Hg^2+^ in 100 mg/L Hg^2+^ solution [115,117]. However, superior values for Hg removal and biosorption capacities obtained herein for fungal isolates from the Turda site, investigated as live biomass, are likely due to the active metabolism of the cells. This facilitates supplementary intracellular uptake, unlike powdered biosorbents. Living fungal cells recovered from contaminated substrates have biosorption capacities in Hg-contaminated medium, in the 0.017–40.2 mg/g Hg^2+^ range [80,88,112,118]. Finally, the Hg^2+^ bioremediation potential from highly contaminated aqueous substrate was demonstrated for five species which were isolated and identified in this study. *Didymella glomerata* isolate was able to develop sufficient biomass to perform 97% Hg^2+^ removal from 100 mg/L aqueous solution. This is important considering the cost of constituents that might be needed for scaling up this type of biosorbent material. Advanced analyses of these biomasses should establish the specificity for Hg removal, optimal removal conditions, treatment efficacy for biomass recovery, and number of cycles for usage at a satisfactory removal capacity. Additionally, future research should focus on molecular resistance mechanisms (membrane and vacuolar transporters, metal chelators, etc.) of these fungal isolates. The molecular determinants of fungal HM resistance can be used to aid mycoremediation as a reliable technology.

## 5. Conclusions

The HMs resistance of fungi, isolated from the plant rhizosphere grown on a highly Hg-contaminated site of a decommissioned chloralkali facility, was in accordance with the ecological risk index for the elements concerned (Hg > Pb > Zn > Cu > Cd). Ascomycota was the dominant phylum in terms of diversity, abundance, multiple HMs, and increased Hg resistance. The detection of Mucoromycota isolates sensitive to Hg and Cu and moderately Zn-resistant suggested that they are likely involved in symbiotic relationships with resident plants. Thus, the association of native HM-resistant fungal isolates with plant-symbiotic ones was emphasized in the development of in situ microbe-assisted phytoremediation of contaminated soil. New species or genera that were previously un-related to Hg-contaminated substrates were identified. Fungi taxonomy reflected their responses to HMs stress, although isolate-specific resistance patterns also occurred. *Fusarium oxysporum*, *Sarocladium kiliense*, *Didymella glomerata*, *Phoma costaricensis*, and *Cladosporium* sp. isolates showed important MICs for the studied elements, as well as efficient Hg^2+^ removal (up to 97%) and biosorption capacities (up to 54.9 mg/g). The characterization and improvement of the novel biosorbent materials should be further explored for industrial water decontamination. Nevertheless, the high degree of fungal resistance for the spectrum of HMs assessed calls for additional research to identify, isolate, and characterize key molecular metal resistance mechanisms. This knowledge would broaden the understanding of HMs detoxification mechanisms in filamentous fungi and contribute to the progress of bioremediation strategies.

## Figures and Tables

**Table 1:** Summary statistics of Cd, Cu, Hg, Pb, and Zn (mg/kg) in rhizosphere soil samples by portable X-ray fluorescence (PXRF) spectrometry for polluted soil in Turda, Romania.

	Hg	Pb	Cd	Cu	Zn
Minimum	35	54	<LOD ^a^	40	226
Maximum	4530	516	<LOD ^a^	1699	2198
Mean	1195	283	- ^c^	586	661
Median	756	266	- ^c^	482	487
SD	1107	125	- ^c^	456	477
Skewness	1.70	0.19	- ^c^	0.98	2.11
Kurtosis	3.35	−0.47	- ^c^	0.53	5.03
Sensitive soil threshold alert ^b^	1	50	3	100	300
Sensitive soil threshold intervention ^b^	2	100	5	200	600
Industrial soil threshold alert ^b^	4	150	5	250	700
Industrial soil threshold intervention ^b^	10	1000	10	500	1500

^a^ LOD—limit of detection, 5 mg/kg Cd; ^b^ Soil threshold values according to Romanian regulations [84]. ^c^ The parameters could not be calculated for Cd, as the values detected in the rhizosphere soil samples were under the LOD.

## Data Availability

Not applicable.

## Supplementary Materials

The following are available online at https://www.mdpi.com/article/10.3390/jof7050386/s1, Table S1: Recovery of metals in NIST certified reference 2711a (Montana II Soil) and correction factors obtained by portable X-ray fluorescence spectrometry; values represent mean (*n* = 5), Table S2: GenBank accession numbers assigned for heavy metal resistant fungal isolates (with Tolerance Index ≥ 0.5 for at least one of the metals assessed) germane to contaminated soils in Turda, Romania, Table S3: Mean growth diameters of fungal isolates tested for Minimum Inhibitory Concentration.
