# Supplementary material for: Heavy Metal-Resistant Filamentous Fungi as Potential Mercury Bioremediators
Source: J Fungi (Basel). 2021 May 14;7(5):386. doi: 10.3390/jof7050386 (PMC8156478; doi:10.3390/jof7050386)
Supplement: Supplementary file 1 [file jof-07-00386-s001.zip › jof-1214632-supplementary.pdf]

Table S1. Recovery of metals in NIST certified reference 2711a (Montana II Soil) and correction factors obtained by portable X-ray fluorescence spectrometry; values represent mean (n = 5).

Element	Certified value Mean±U[a] (mg/kg)	Found value Mean±CI[b] (mg/kg)	Recovery Mean±CI[b] (%)	Correction factor
Cd	54.1 ± 0.5	58.6 ± 5.1	108 ± 9	0.923
Cu	140 ± 2	118 ± 4	85 ± 4	1.186
Hg	7.42 ± 0.18	11.68 ± 1.60	157 ± 13	0.635
Pb	1400 ± 10	1374 ± 11	98 ± 1	1.019
Zn	414 ± 11	356 ± 14	86 ± 4	1.162

[a] U is the expanded uncertainty for 95% confidence level (k=2);
[b] CI is the confidence interval for 95% confidence level (n=5).

Table S2. GenBank accession numbers assigned for heavy metal resistant fungal isolates (with Tolerance Index ≥ 0.5 for at least one of the metals assessed) germane to contaminated soils in Turda, Romania.

Isolate ID	Species	GenBank accession number
P2.1	*Sarocladium kiliense*	MT913526
P2.2	*Sarocladium kiliense*	MT913527
P2.5	*Fusarium oxysporum*	MN519436
P2.7	*Fusarium oxysporum*	MT913528
P2.9	*Penicillium crustosum*	MT913529
P2.10	*Phoma costaricensis*	MT913530
P2.12	*Stagonosporopsis* sp.	MT913531
P2.14	*Fusarium oxysporum*	MT913532
P2.15	*Mortierella alpina*	MT913533
P2.16	*Didymella glomerata*	MT913534
T1	*Mortierella alpina*	MT913535
T2	*Cladosporium* sp.	MT913536
T3	*Fusarium oxysporum*	MT913537
T4	*Mortierella alpina*	MT913538
T8	*Fusarium equiseti*	MT913539
T14	*Lecanicillium* sp.	MT913540
T16	*Mortierella alpina*	MT913541
T21	*Sarocladium kiliense*	MT913542
T23	*Mortierella alpina*	MT913543
T25	*Sarocladium* sp.	MT913544
T27	*Fusarium oxysporum*	MT913545
T28	*Fusarium solani*	MT913546

T30	*Aspergillus* sp.	MT913547
T32	*Cladosporium macrocarpum*	MT913548
T33	*Aspergillus* sp.	MT913549
T34	*Sarocladium kiliense*	MT913550
T36	*Cadophora malorum*	MT913551
T37	*Cadophora malorum*	MT913552
T39	*Sarocladium kiliense*	MT913553
TRD3.2	*Cladosporium* sp.	MT913554
TRD5.5	*Penicillium brevicompactum*	MT913555
TRD5P.6	*Sarocladium kiliense*	MT913556

Table S3. Mean growth diameters of fungal isolates tested for Minimum Inhibitory Concentration.

**Species**	**Isolate**	**Metal**	**Metal concentration (mg/L)**	**Diameter mean (mm) ± SE** (n=3-9)
Fusarium oxysporum	P2.5	Hg	0	82.83 ± 0.40
			80	51.67 ± 0.41
			120	34.93 ± 0.42
			140	6.00 ± 0.00
		Pb	518	33.33 ± 0.25
			1036	16.17 ± 0.10
			1568	6.00 ± 0.00
		Zn	490	51.83 ± 0.84
			653	42.67 ± 0.25
			1176	23.83 ± 0.19
			1569	15.50 ± 0.25
			2092	7.50 ± 0.73
			2353	6.50 ± 0.17
	P2.7	Hg	0	85.00 ± 0.00
			80	85.00 ± 0.00
			120	55.86 ± 1.11
			140	28.75 ± 0.71
			180	24.50 ± 0.93
			200	6.00 ± 0.00
		Pb	518	33.33 ± 0.38
			1036	15.00 ± 2.02
			1568	6.00 ± 0.00
		Zn	490	49.33 ± 0.35
			653	41.00 ± 0.44
			1176	22.50 ± 0.17
			1569	15.00 ± 0.45
			1830	11.83 ± 0.19
			2092	6.33 ± 0.10

Sarocladium kiliense	P2.2	Hg	0	32.25 ± 0.10
			40	30.17 ± 0.10
			160	9.17 ± 0.16
			200	6.33 ± 0.16
		Cd	63.5	29.50 ± 0.41
			127	20.83 ± 0.08
			254	10.00 ± 0.00
			381	6.00 ± 0.00
		Pb	518	12.83 ± 0.08
			1036	7.00 ± 0.00
			2072	6.33 ± 0.16
	TRD5P6	Hg	0	43.58 ± 0.31
			30	38.67 ± 0.19
			80	26.50 ± 0.17
			160	11.30 ± 0.19
		Cd	168	28.00 ± 0.00
			224	23.00 ± 0.33
			281	18.92 ± 0.06
			337	15.00 ± 0.29
			337	10.50 ± 0.17
Didymella glomerata	P2.16	Hg	0	74.83 ± 0.35
			80	45.67 ± 1.67
			160	14.33 ± 0.19
			200	7.17 ± 0.67
Phoma costaricensis	P2.10	Hg	0	55.17 ± 1.18
			30	53.17 ± 1.18
			80	41.00 ± 1.8
			160	6.00 ± 0.00
Cladosporium sp.	TRD3.2	Hg	0	31.17 ± 1.49
			10	29.50 ± 0.44
			30	26.17 ± 1.40
			80	24.67 ± 0.19
			160	6.00 ± 0.00
